# Physical activity and Alzheimer’s disease risk across genetic susceptibility: a prospective UK Biobank study using accelerometer data

**DOI:** 10.1007/s00415-025-13524-z

**Published:** 2025-12-02

**Authors:** Seunghee Na, Kenneth Muir, Artitaya Lophatananon, Yicong Huang, Jongin Lee

**Affiliations:** 1 Department of Neurology, Incheon St. Mary’s Hospital, College of Medicine, The Catholic University of Korea, Seoul, Republic of Korea; 2 Division of Population Health, Health Services Research and Primary Care, Faculty of Biology, Medicine and Health, School of Health Sciences, The University of Manchester, Manchester, UK; 3 Department of Occupational and Environmental Medicine, Seoul St. Mary’s Hospital, College of Medicine, The Catholic University of Korea, Seoul, Republic of Korea

**Keywords:** Alzheimer's disease, Physical activity, UK Biobank, APOE gene

## Abstract

**Objective:**

Physical activity (PA) has been associated with reduced Alzheimer’s disease (AD) risk, but whether protective effects vary across genetic risk levels remains unclear. Previous studies were limited by self-reported PA measures and simplified genetic models. In this study, we aimed to examine the association between accelerometer-measured physical activity and the risk of incident AD in a large population-based cohort, and to explore potential interactions between PA and polygenic risk scores for AD.

**Methods:**

We analyzed 93,578 UK Biobank participants aged 40–70 years with accelerometer data and genome-wide genotyping. PA was measured continuously (milligravity, mg) and dichotomized at the optimal point from maximally selected rank statistics. Genetic risk was assessed using polygenic risk scores (PRS) and APOE ε4 status. Cox models estimated hazard ratios for incident AD across genetic risk strata during median 15.5-year follow-up.

**Results:**

Among 401 AD cases, high PA reduced risk by 48% (HR 0.517; 95% CI 0300–0.891), while high PRS increased risk nearly twofold (HR 2.423; 95% CI 1.757–3.343). PA’s protective association remained consistent across all PRS and APOE ε4 strata. No significant multiplicative or additive interaction was found between PA and genetic risk (RERI = − 0.566, 95% CI − 4.574–3.441). Dose–response analysis revealed maximum benefit with optimal threshold at 21.7 mg corresponding to light-intensity activity.

**Conclusion:**

Objectively measured PA substantially reduces AD risk regardless of genetic predisposition. Even light-intensity activity provides meaningful protection, supporting PA as a broadly applicable preventive strategy across all genetic risk levels.

**Electronic supplementary material:**

The online version of this article (10.1007/s00415-025-13524-z) contains supplementary material, which is available to authorized users.

## Introduction

Alzheimer’s disease (AD) is the most common cause of dementia and a leading contributor to disability and dependency among older adults worldwide [12]. With rapid global population aging, particularly in high- and middle-income countries, the burden of AD is expected to increase substantially over the coming decades [9]. This growing public health challenge not only impacts patients and families, but also places enormous strain on healthcare and long-term care systems. Identifying modifiable risk factors and early preventive strategies is therefore of critical importance.

While aging remains the most prominent risk factor for AD, genetic susceptibility plays a key role in determining individual risk. The apolipoprotein E (APOE) ε4 allele is the most well-established genetic variant associated with increased AD risk, but it does not fully explain the heritability of the disease [2]. Recent genome-wide association studies (GWAS) have identified multiple loci with modest effects, highlighting the polygenic nature of AD [1].

Given the complexity of its genetic architecture, polygenic risk scores (PRS) which aggregate the effects of numerous AD-associated single-nucleotide polymorphisms (SNPs) have emerged as a powerful tool for stratifying individuals by inherited risk [3]. PRS allows for more comprehensive modeling of genetic liability than single-gene approaches and can help identify high-risk individuals even in the absence of APOE ε4 [14].

In parallel, physical activity (PA) has been consistently associated with reduced risk of cognitive decline and AD in observational studies [23]. Mechanistic evidence suggests that PA may improve neurovascular health, reduce inflammation, and enhance neuroplasticity [7]. As a modifiable lifestyle factor, PA represents a promising target for public health interventions aimed at delaying or preventing AD onset [10].

Recent studies have explored whether the protective effects of PA vary according to genetic susceptibility to dementia, with mixed findings regarding gene-environment interactions [27]. However, these studies have primarily relied on self-reported physical activity measures, which may be subject to recall bias and measurement error. It remains unclear whether such interactions exist when using objective, accelerometer-based measurements of physical activity and comprehensive polygenic risk assessment. Understanding the interaction between genetic predisposition and modifiable behaviors such as PA may yield important insights for personalized prevention strategies. In this study, we aimed to examine the association between accelerometer-measured physical activity and the risk of incident AD in a large population-based cohort, and to explore potential interactions between PA and polygenic risk scores for AD.

## Methods

### Study population

**Fig. 1:**
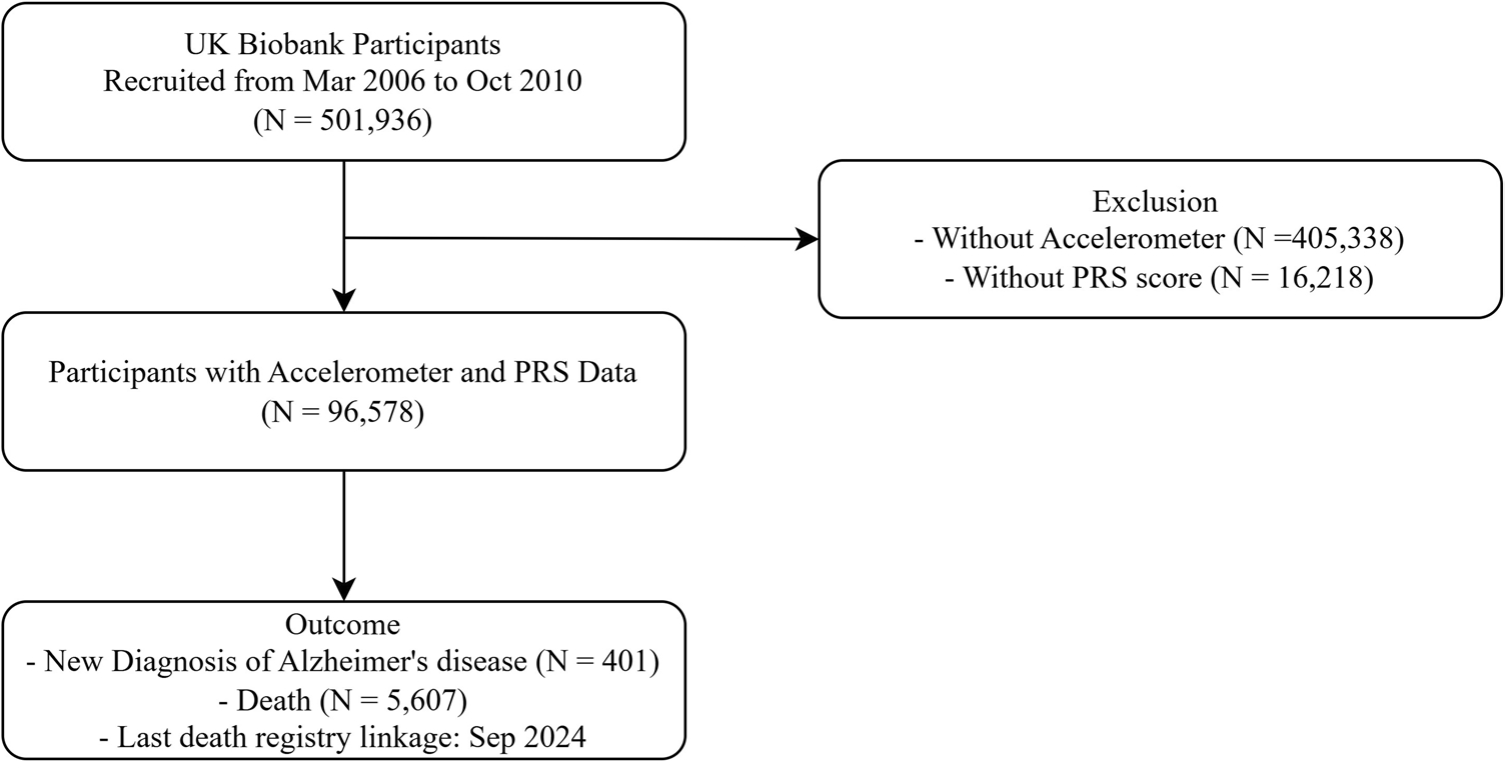
Flow diagram of participant inclusion and exclusion in the study cohort

We utilized data from the UK Biobank, a large-scale prospective cohort study comprising 501,936 individuals aged 40–69 years at recruitment between 2006 and 2010. For the present analysis, we included participants with complete data on wrist-worn accelerometer-based physical activity, PRS for AD, and relevant covariates. Participants with missing or poor-quality accelerometer data, as indicated by UK Biobank calibration and wear-time flags, were excluded. After complete-case exclusion, the final analytic sample consisted of 95,578 individuals (Fig. 1).

### Exposure assessment

PA was objectively assessed using triaxial wrist-worn accelerometers (Axivity AX3), with daily activity quantified as the average vector magnitude in milligravity (mg). This continuous measure (UK Biobank variable: 90012) reflects overall daily activity levels. For categorical analyses, PA was dichotomized into “Low” and “High” groups based on the sample median value. The median threshold used for classification was 27.05 mg.

Genetic susceptibility to AD was assessed using the standardized PRS in UK Biobank [24]. Participants were categorized into “Low” and “High” PRS groups based on the sample median value. The expression of AD is known to be strongly associated with a single genotype of the APOE gene [5]. The subtypes of the APOE gene were determined based on the presence of rs429358 and rs7412 in participants of the UK Biobank.

### Outcome ascertainment

The primary outcome was a diagnosis of AD, as defined by the UK Biobank’s algorithmically defined outcome (ADO) data [6]. Cases of AD were identified based on the earliest occurrence of a diagnosis from any of the following sources: hospital inpatient records, national death registries, or self-reporting at the baseline assessment. AD cases were identified using UK Biobank’s ADO for Alzheimer’s disease (Data-Field 42020), which captures diagnoses from hospital inpatient records (ICD-10 codes F00.x and G30.x), death certificates, and self-reports at baseline assessment. The ADO algorithm has been validated in UK Biobank participants with a positive predictive value of 71.4% for Alzheimer’s disease specifically when combining all data sources [26]. Follow-up time was calculated from the baseline assessment date to the earliest AD diagnosis date, death date, or administrative censoring date on 30 September 2024. To minimize the impact of preclinical or undiagnosed AD at baseline, participants diagnosed with AD within 4 years of their initial assessment were excluded. As a result, 401 newly onset AD patients were included in this study.

### Covariates

We adjusted for a comprehensive set of demographic, socioeconomic, and health-related covariates known to be associated with both physical activity and AD risk. Age was categorized into four groups: 30–49, 50–59, 60–69, and 70 years or older. Gender and ethnicity (White vs. Non-White) were included as basic demographic variables. Socioeconomic status (SES) was derived from the Townsend deprivation index and classified into tertiles (low, middle, and high SES). Educational attainment was categorized as none/other, secondary, or university-level education.

Lifestyle factors included smoking status (never, former, current, or no response) and alcohol consumption (non-drinker, light, heavy, or no response). Sleep duration was grouped into ≥ 7 h, < 7 h, or missing. Body mass index (BMI) was classified into underweight, normal, overweight, obese, or no response, based on measured height and weight. Additionally, the presence of hypertension and diabetes at baseline was included as a binary variable (yes/no), based on diagnosis records prior to the UK Biobank assessment date. All Cox models were adjusted for PRS-specific genetic principal components to account for population stratification [24].

### Statistical analysis

Cox proportional hazards models were used to estimate hazard ratios (HRs) and 95% confidence intervals (CIs) for the association of PA and APOE/PRS with the risk of incident AD. Both unadjusted and fully adjusted models were fitted, with the latter including all covariates listed above. A multiplicative interaction term between PA and APOE/PRS was included to evaluate potential effect modification. Physical activity was modeled in three complementary ways in the Cox models: (1) dichotomized at the sample median; (2) as a continuous variable presenting HRs change by each standard deviation (SD); and (3) per 10 mg increase in average acceleration. The proportional hazards assumption was evaluated using Schoenfeld residuals. The assumption was satisfied for PA (*p* = 0.84), PRS (*p* = 0.81), APOE ε4 carrier status (*p* = 0.38), and the global test (*p* = 0.12). The findings from the Fine–Gray model, which defined mortality as a competing risk, were also explicitly stated for the primary analysis results.

To examine additive interaction between PA and APOE/PRS, we created a four-level exposure variable combining PRS (Low/High) and PA (Low/High), using the Low PRS/High PA group as the reference. Regarding APOE gene grouping, subjects were classified according to the presence or absence of the risk factor ε4 carrier. Measures of additive interaction—including the relative excess risk due to interaction (RERI), attributable proportion (AP), and synergy index (SI)—were calculated with 95% CIs derived via the delta method.

Restricted cubic spline (RCS) regression models were applied to assess potential non-linear associations between continuous PA and AD risk, using the rms package. A spline function with four knots was fitted, and the resulting hazard functions were visualized with and without 95% confidence intervals.

All statistical analyses were conducted using R (version 4.4.2). A two-sided *p* value < 0.05 was considered statistically significant.

## Results

### Baseline characteristics

The analytic sample included 96,578 participants. PRS groups were approximately equally distributed. Stratification by PA revealed more pronounced differences. Participants in the High PA group were more likely to be younger and female, with a greater proportion having university-level education. The Low PA group, by contrast, had a higher proportion of participants aged 60–69 years, more obesity, and higher prevalence of cardiometabolic comorbidities, such as hypertension and diabetes. Lifestyle behaviors also differed: current smoking was more frequent in the Low PA group, while the High PA group had more heavy drinkers. Individuals in the high PA group were more likely to meet PA guidelines (52.1% vs. 39.1%) and maintain a normal BMI (47.1% vs. 30.4%). The prevalence of APOE ε4 carrier and AD incidence was significantly lower in the high PA group. (Table 1).

**Table 1:** Baseline characteristics of the study population according to physical activity level

	Low PA	High PA	*p* value
	(*n* = 48,266)	(*n* = 48,312)	
Age			
30–49	8014 (16.6)	14,612 (30.2)	< 0.001
50–59	16,234 (33.6)	18,746 (38.8)	
60–69	23,775 (49.3)	14,871 (30.8)	
70+	243 (0.5)	83 (0.2)	
Sex			
Male	22,543 (46.7)	19,658 (40.7)	< 0.001
Female	25,723 (53.3)	28,654 (59.3)	
Ethnicity			
White	46,793 (96.9)	46,471 (96.2)	< 0.001
Non-White	1473 (3.1)	1841 (3.8)	
Education			
None/Other	10,297 (21.5)	8098 (16.9)	< 0.001
Secondary	17,571 (36.6)	18,539 (38.6)	
University	20,086 (41.9)	21,417 (44.6)	
Smoking status			
Never	26,459 (54.8)	28,559 (59.1)	< 0.001
Previous	17,894 (37.1)	16,731 (34.6)	
Current	3777 (7.8)	2900 (6.0)	
No answer	136 (0.3)	122 (0.3)	
Alcohol status			
Non-drinker	8050 (16.7)	6591 (13.6)	< 0.001
Light drinker	17,401 (36.1)	17,304 (35.8)	
Heavy drinker	22,772 (47.2)	24,379 (50.5)	
No answer	43 (0.1)	38 (0.1)	
Daily sleeping time			
≥ 7 h	37,513 (77.7)	37,624 (77.9)	0.020
< 7 h	10,586 (21.9)	10,568 (21.9)	
No answer	167 (0.3)	120 (0.2)	
Body mass index			
Normal	14,650 (30.4)	22,732 (47.1)	< 0.001
Underweight	177 (0.4)	368 (0.8)	
Overweight	20,722 (42.9)	18,966 (39.3)	
Obese	12,569 (26.0)	6176 (12.8)	
No answer	148 (0.3)	70 (0.1)	
Hypertension			
No	34,123 (70.7)	40,104 (83.0)	< 0.001
Yes	14,143 (29.3)	8208 (17.0)	
Diabetes mellitus			
No	45,752 (94.8)	47,439 (98.2)	< 0.001
Yes	2514 (5.2)	873 (1.8)	
IPAQ recommendation			
Yes	18,884 (39.1)	25,170 (52.1)	< 0.001
No	20,420 (42.3)	15,450 (32.0)	
No answer	8962 (18.6)	7692 (15.9)	
APOE ε4 carrier			
Non-carrier	36,169 (76.7)	35,745 (75.4)	< 0.001
Carrier	11,015 (23.3)	11,635 (24.6)	
AD incidence			
No	47,990 (99.4)	48,187 (99.7)	< 0.001
Yes	276 (0.6)	125 (0.3)	

Mean age was similar across PRS groups though the high PRS group had a slightly higher proportion of participants aged 30–59. Gender, ethnicity distributions, and education level were comparable. Alcohol consumption, smoking status, and average sleeping duration did not differ significantly. The high PRS group included a slightly lower proportion of participants with normal BMI and a slightly lower proportion with obesity (19.0% vs. 19.7%). Clinical comorbidities including hypertension (22.8% vs. 23.5%) and diabetes (3.4% vs. 3.6%) were modestly less prevalent in the high PRS group. Furthermore, participants in the high PRS group were more likely to meet PA guidelines (46.1% vs. 45.5%) and had a slightly higher rate of high accelerometer-measured PA (50.6% vs. 49.7%). Notably, the prevalence of APOE ε4 carrier and AD incidence were significantly higher in the high PRS group (Table 2).

**Table 2:** Baseline characteristics of the study population according to polygenic risk score (PRS) groups

	Low PRS	High PRS	*p* value
	(*n* = 47,743)	(*n* = 46,493)	
Age			
30–49	11,091 (23.2)	10,964 (23.6)	0.011
50–59	17,117 (35.9)	16,997 (36.6)	
60–69	19,371 (40.6)	18,380 (39.5)	
70+	164 (0.3)	152 (0.3)	
Sex			
Male	20,943 (43.9)	20,374 (43.8)	0.896
Female	26,800 (56.1)	26,119 (56.2)	
Ethnicity			
White	46,092 (96.5)	44,961 (96.7)	0.172
Non-White	1651 (3.5)	1532 (3.3)	
Education			
None/Other	9226 (19.4)	8782 (18.9)	0.219
Secondary	17,882 (37.6)	17,468 (37.7)	
University	20,510 (43.1)	20,129 (43.4)	
Smoking status			
Never	27,081 (56.7)	26,592 (57.2)	0.252
Previous	17,217 (36.1)	16,601 (35.7)	
Current	3312 (6.9)	3193 (6.9)	
No answer	133 (0.3)	107 (0.2)	
Alcohol status			
Non-drinker	7232 (15.1)	7007 (15.1)	0.542
Light drinker	17,077 (35.8)	16,752 (36.0)	
Heavy drinker	23,394 (49.0)	22,705 (48.8)	
No answer	40 (0.1)	29 (0.1)	
Daily sleeping time			
≥ 7 h	37,212 (77.9)	36,122 (77.7)	0.520
< 7 h	10,402 (21.8)	10,233 (22.0)	
No answer	129 (0.3)	138 (0.3)	
Body mass index			
Normal	18,413 (38.6)	18,131 (39.0)	0.001
Underweight	233 (0.5)	293 (0.6)	
Overweight	19,601 (41.1)	19,176 (41.2)	
Obese	9391 (19.7)	8813 (19.0)	
No answer	105 (0.2)	80 (0.2)	
Hypertension			
No	36,511 (76.5)	35,896 (77.2)	0.008
Yes	11,232 (23.5)	10,597 (22.8)	
Diabetes mellitus			
No	46,012 (96.4)	44,924 (96.6)	0.038
Yes	1731 (3.6)	1569 (3.4)	
Accelerometer			
Low	24,038 (50.3)	22,968 (49.4)	0.004
High	23,705 (49.7)	23,525 (50.6)	
IPAQ recommendation			
Yes	21,732 (45.5)	21,447 (46.1)	0.011
No	18,005 (37.7)	17,096 (36.8)	
No answer	8006 (16.8)	7950 (17.1)	
APOE ε4 carrier			
Non-carrier	45,754 (95.8)	25,913 (55.7)	< 0.001
Carrier	1989 (4.2)	20,580 (44.3)	
AD incidence			
No	47,665 (99.8)	46,184 (99.3)	< 0.001
Yes	78 (0.2)	309 (0.7)	

### Cox regression

In the crude model, both PA and PRS showed significant associations with incident AD. Participants in the High PA group had a markedly lower risk of AD compared to those in the Low PA group (HR 0.507, 95% CI 0.295–0.872, *p* < 0.001). Conversely, individuals with High PRS had a markedly increased risk of AD compared to those with Low PRS (HR 3.861, 95% CI 2.877–5.180, *p* < 0.001).

These associations remained robust after adjustment for age as the time scale, sex, socioeconomic status, education, ethnicity, smoking, alcohol consumption, sleep duration, BMI, hypertension, and diabetes. In the fully adjusted model, high PA was associated with a 48.3% lower risk of AD (HR 0.517, 95% CI 0.300–0.891, *p* < 0.001), while high PRS continued to be a strong risk factor (HR 2.423, 95% CI 1.757–3.343, *p* < 0.001). Each SD increase in PA was associated with a 29% lower AD risk (HR 0.823, 95% CI 0.721–0.938, *p* < 0.001), and each 10 mg increase with a 34% lower risk (HR 0.791, 95% CI 0.676–0.926, *p* < 0.001).

Accounting for competing risks of death, the sub-distribution hazard ratios showed consistent protective effects: High PA (sHR 0.327, 95% CI 0.189–0.565), continuous PA (sHR 0.604, 95% CI 0.524–0.696), and per 10 mg increase (sHR 0.546, 95% CI 0.46–0.648), while high PRS remained a significant risk factor (sHR 2.387, 95% CI 1.726–3.302).

Other covariates, such as older age, male, low educational attainment, obesity, and presence of hypertension or diabetes, were associated with increased AD risk, though their effect sizes were generally smaller than those of PA and PRS (Table 3).

**Table 3:** Hazard ratios and subdistribution hazard ratios for Alzheimer’s disease according to polygenic risk score and physical activity levels

Variable	Incidence		Crude			Adjusted*			Adjusted*		
	%	/10,000PY	HR	95% CI Lower	95% CI Upper	HR	95% CI Lower	95% CI Upper	sHR	95% CI Lower	95% CI Upper
Physical activity											
Low	0.567	3.71	(Ref)			(Ref)			(Ref)		
High	0.258	1.67	0.507	0.295	0.872	0.517	0.300	0.891	0.327	0.189	0.565
Continuous	–	–	0.821	0.725	0.931	0.823	0.721	0.938	0.604	0.524	0.696
Per 10 mg	–	–	0.790	0.680	0.918	0.791	0.676	0.926	0.546	0.460	0.648
Polygenic risk score											
Low	0.156	1.02	(Ref)			(Ref)			(Ref)		
High	0.665	4.33	3.861	2.877	5.180	2.423	1.757	3.343	2.387	1.726	3.302
PA:PRS interaction	–	–	1.679	0.93	3.032	1.636	0.906	2.955	1.621	0.898	2.926

^*^Adjusted for age as the time scale, sex, Townsend deprivation index, ethnicity, education, smoking status, alcohol status, sleeping time per day, body mass index, prevalence of hypertension/diabetes, and genetic principal components

### Interaction analysis

Individuals were categorized into four groups according to combinations of PRS and PA levels. Among non-carriers of the APOE ε4 allele, compared to those with low PRS and high PA, participants with low PRS and low PA had a significantly increased risk of Alzheimer’s disease (HR 1.940, 95% CI 1.081–3.479). Those with high PRS and high PA had a similarly elevated risk (HR 4.478, 95% CI 2.458–8.157), while participants with high PRS and low PA showed the highest risk (HR 4.852, 95% CI 2.734–8.607). In APOE ε4 carriers, compared to those with low PRS and high PA, the highest risk was observed in individuals with high PRS and low PA (HR 3.736, 95% CI 0.923–15.129), followed by those with high PRS and high PA (HR 3.053, 95% CI 0.748–12.463) and low PRS and low PA (HR 2.176, 95% CI 0.451–10.498). No meaningful additive interaction between physical activity and polygenic risk for Alzheimer’s disease was observed on the additive scale. Specifically, the relative excess risk due to interaction (RERI) was − 0.566 (95% CI − 4.574–3.441) in non-carriers and − 0.493 (95% CI − 8.075–7.090) in ε4 carriers, suggesting no synergistic effect beyond the individual contributions of high polygenic risk and low physical activity. Similarly, the attributable proportion due to interaction (AP) was − 0.117 (95% CI − 0.943–0.709) in non-carriers and − 0.132 (95% CI − 2.162–1.898) in ε4 carriers. The synergy index (SI) was 0.872 (95% CI 0.450–1.690) and 0.847 (95% CI 0.173–4.150), indicating no evidence of positive additive interaction between these two factors in increasing Alzheimer’s risk (Table 4).

**Table 4:** Interaction between APOE gene, PRS and physical activity on the risk of Alzheimer’s disease

(a) Adjusted* hazard ratios (HRs) and 95% confidence intervals by combined exposure groups							
		Incidence				Incidence	
Group in APOE ε4 non-carrier	HR [95% CI]	%	/10,000PY	Group in APOE ε4 carrier	HR [95% CI]	%	/10,000PY
PRS low + high PA (ref)	1.000 (ref)	0.067	0.43	PRS low + PA high (ref)	1.000 (ref)	0.197	1.28
PRS low + low PA	1.940 [1.081–3.482]	0.219	1.43	PRS low + PA low	2.176 [0.451–10.502]	0.728	4.74
PRS high + high PA	4.478 [2.458–8.160]	0.295	1.91	PRS high + PA high	3.053 [0.748–12.468]	0.637	4.13
PRS high + low PA	4.852 [2.754–8.548]	0.528	3.46	PRS high + PA low	3.736 [0.923–15.131]	1.350	8.88

(b) Additive interaction indices							
Index	Estimate [95% CI]			Index	Estimate [95% CI]		
RERI	− 0.566 [− 4.574–3.441]			RERI	− 0.493 [− 8.075–7.090]		
Attributable proportion (AP)	− 0.117 [− 0.943–0.709]			Attributable proportion (AP)	− 0.132 [− 2.162–1.898]		
Synergy index (SI)	0.872 [0.450–1.690]			Synergy index (SI)	0.847 [0.173–4.150]		

^*^Adjusted for age as the time scale, sex, Townsend deprivation index, ethnicity, education, smoking status, alcohol status, sleeping time per day, body mass index, prevalence of hypertension/diabetes, and genetic principal components

Among individuals with high physical activity, the risk of Alzheimer’s disease remained low in the bottom two quintiles of PRS. Participants in the third quintile (HR 1.484, 95% CI 0.704–3.128) began to show a gradual increase in risk, while those in the fourth (HR 3.678, 95% CI 1.979–6.831) and fifth quintiles (HR 7.638, 95% CI 4.331–13.474) exhibited substantially elevated risks (Table 5). When the same interaction analysis was conducted based on whether the participants met the IPAQ physical activity recommendations, the clear correlations observed in each group when evaluated using an accelerometer became ambiguous (Table 6).

**Table 5:** Risk of Alzheimer’s disease according to physical activity status based on PRS quartiles and APOE genotype

	PA low*	%	/10,000PY	PA high*	%	/10,000PY
PRS						
PRS quintile 1	1.000 (ref)	0.16	1.04	0.584 [0.212–1.610]	0.05	0.35
PRS quintile 2	2.028 [1.091–3.770]	0.32	2.10	1.059 [0.463–2.426]	0.10	0.62
PRS quintile 3	2.101 [1.134–3.892]	0.33	2.16	1.484 [0.704–3.126]	0.14	0.90
PRS quintile 4	3.596 [2.022–6.396]	0.56	3.67	3.678 [1.979–6.835]	0.34	2.20
PRS quintile 5	9.822 [5.754–16.765]	1.44	9.46	7.638 [4.331–13.469]	0.66	4.24
APOE gene						
ε4 non-carrier	1.000 (ref)	0.33	2.17	0.755 [0.542–1.052]	0.15	0.97
ε4 carrier	4.133 [3.235–5.280]	1.29	8.47	3.236 [2.388–4.384]	0.60	3.87
ε3/ε3	1.00 (ref)	0.29	1.92	0.783 [0.513–1.197]	0.14	0.90
ε2 carrier (ε2/ε2 or ε2/ε3)	0.579 [0.289–1.159]	0.17	1.14	0.549 [0.221–1.364]	0.10	0.62
ε4 hetero (ε2/ε4 or ε3/ε4)	4.013 [2.972–5.417]	1.12	7.35	3.300 [2.312–4.711]	0.55	3.53
ε4/ε4	13.804 [8.827–21.587]	3.31	22.01	7.806 [4.108–14.835]	1.19	7.72

^*^Adjusted for age as the time scale, sex, Townsend deprivation index, ethnicity, education, smoking status, alcohol status, sleeping time per day, body mass index, prevalence of hypertension/diabetes, and genetic principal components

**Table 6:** Risk of Alzheimer’s disease according to physical activity assessed using IPAQ questionnaires based on PRS quartiles and APOE genotype

(a) Adjusted* hazard ratios (HRs) and 95% confidence intervals by combined exposure groups							
		Incidence					
Group in APOE ε4 non-carrier	HR [95% CI]	%	/10,000	Group in APOE ε4 carrier	HR [95% CI]	%	/10,000
PRS low + high PA (ref)	1.00 (ref)	0.15	0.98	PRS low + PA high (ref)	1.00 (ref)	0.63	2.64
PRS low + low PA	0.665 [0.358–1.234]	0.11	0.68	PRS low + PA low	0.809 [0.202–3.240]	0.41	4.14
PRS high + high PA	3.099 [1.989–4.828]	0.44	2.89	PRS high + PA high	1.267 [0.553–2.906]	0.87	5.68
PRS high + low PA	2.695 [1.640–4.430]	0.37	2.18	PRS high + PA low	1.399 [0.604–3.243]	0.81	5.31

(b) Additive interaction indices							
Index	Estimate [95% CI]			Index	Estimate [95% CI]		
RERI	− 0.068 [− 2.031–1.894]			RERI	0.323 [− 1.613–2.260]		
Attributable proportion (AP)	− 0.025 [− 0.753–0.703]			Attributable proportion (AP)	0.231 [− 1.160–1.622]		
Synergy index (SI)	0.961 [0.147–6.303]			Synergy index (SI)	5.256 [0.001–18533.683]		

^*^Adjusted for age as the time scale, sex, Townsend deprivation index, ethnicity, education, smoking status, alcohol status, sleeping time per day, body mass index, prevalence of hypertension/diabetes, and genetic principal components

### Splining and cutoff for physical activity

**Fig. 2:**
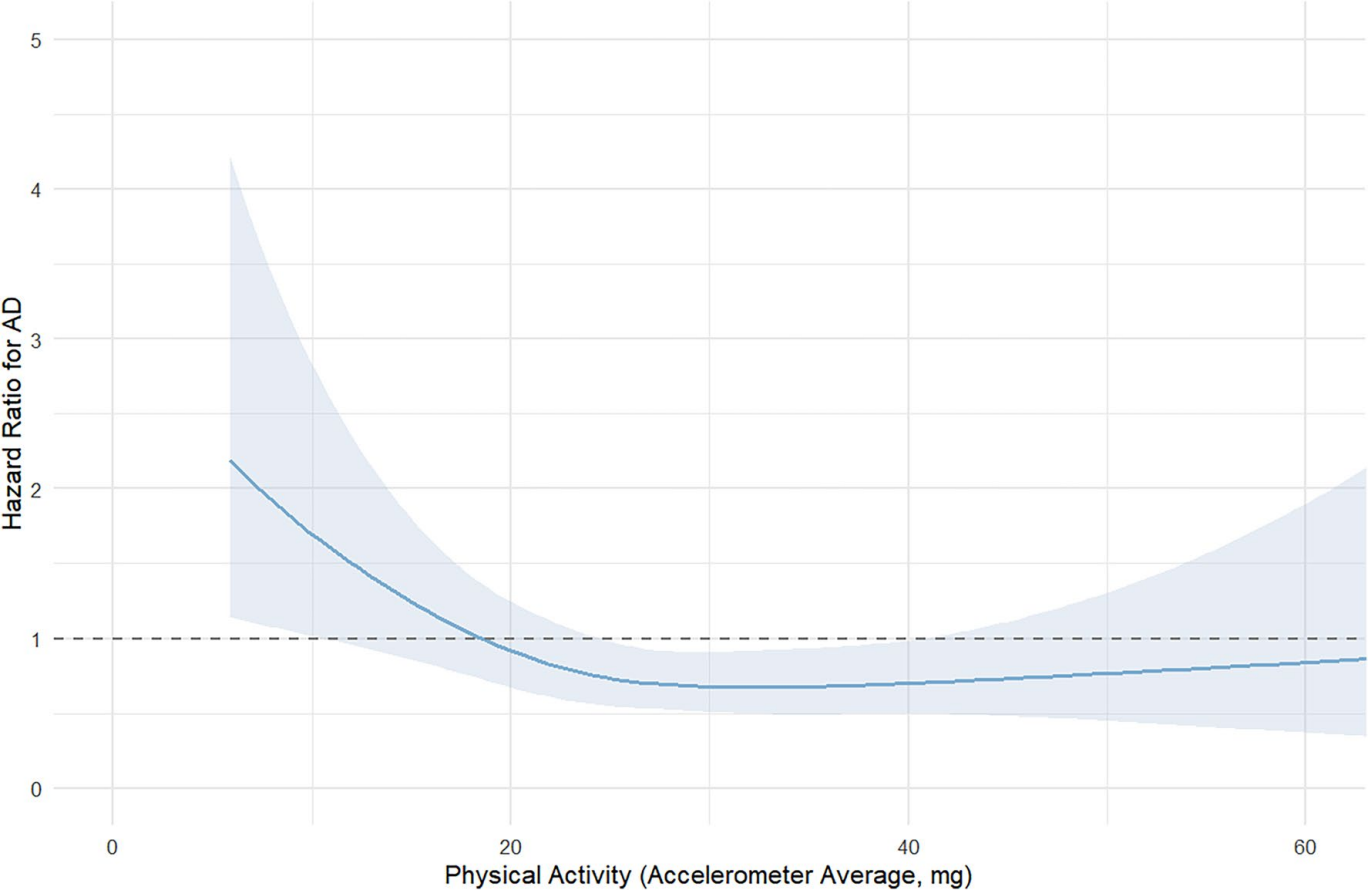
Nonlinear association between accelerometer-measured physical activity and Alzheimer’s disease risk

**Fig. 3:**
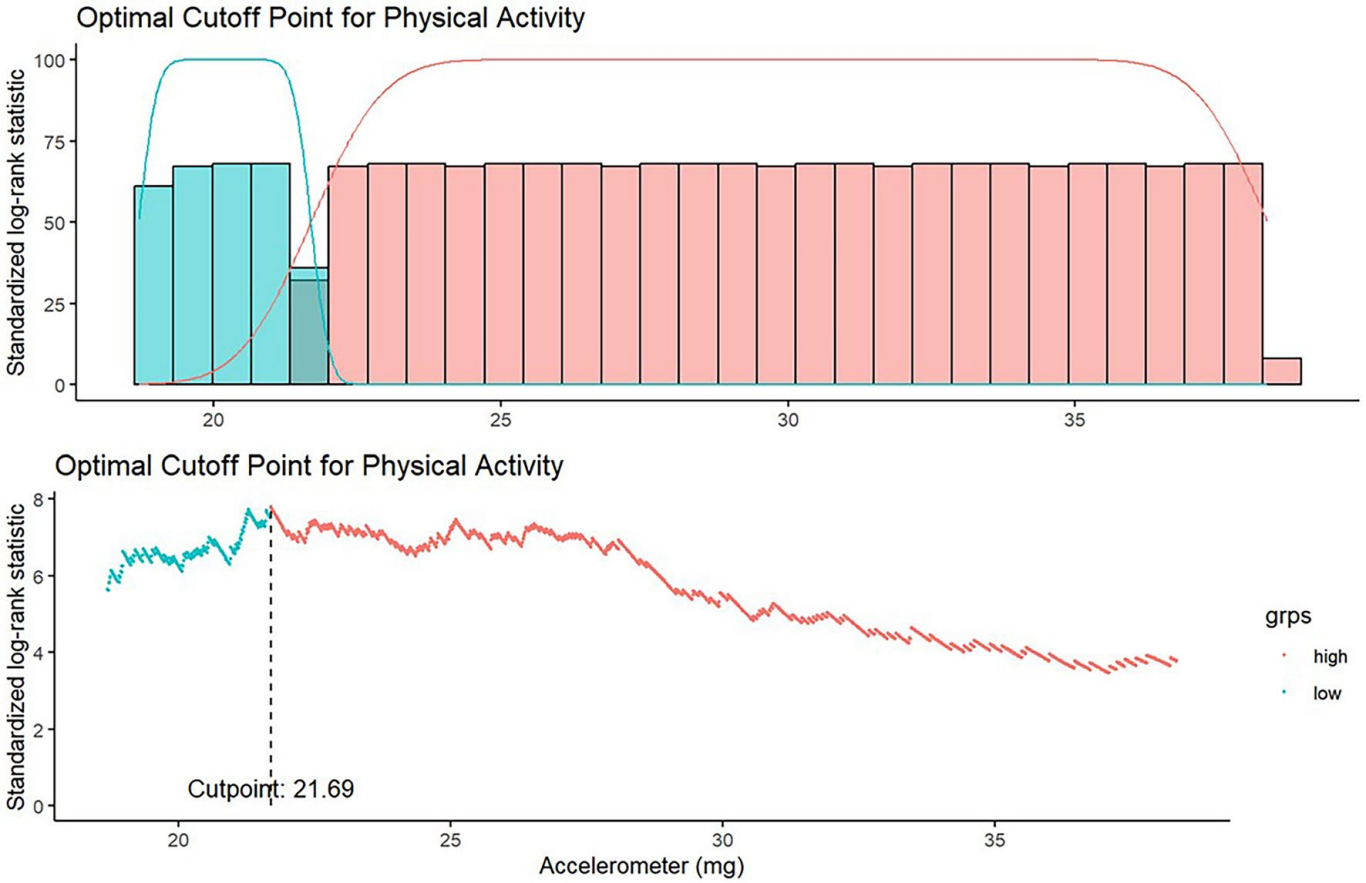
Optimal cutoff for physical activity using maximally selected rank statistics

A restricted cubic spline model was used to assess the non-linear association between continuous accelerometer-measured PA and AD incidence. After adjusting for covariates, the hazard ratio for AD declined steeply with increasing PA levels up to approximately 20–25 mg, after which the curve plateaued (Fig. 2). The most prominent risk reduction was observed between 0 and 25 mg. To determine an optimal threshold for binary classification, a maximally selected rank statistics approach identified 21.69 mg as the cutpoint that best separated high and low-risk groups for AD (Fig. 3).

## Discussion

In this large prospective cohort study, we found that objectively measured PA was associated with a substantially reduced risk of AD across all levels of genetic susceptibility. Higher accelerometer-measured PA was linked to lower AD incidence, even among individuals carrying high polygenic risk or APOE ε4 alleles. Conversely, a high PRS for AD was a strong independent predictor of future dementia. Notably, we did not observe a significant multiplicative or additive interaction between PA and genetic risk, suggesting independent effects. These findings support the utility of PA as a modifiable factor in AD prevention across all genetic backgrounds.

Participants with high PRS had a 3.9-fold higher hazard of developing AD compared to those with low PRS. This is higher than previously reported estimates from population-based studies, where hazard ratios ranged from 1.1 to 1.4 between high and low PRS groups [14, 16]. Differences in PRS construction, inclusion of the APOE region, and cohort characteristics likely contribute to this discrepancy. Our PRS included APOE and was applied to a relatively healthy, middle-aged cohort. The high PRS group had a substantially greater proportion of APOE ε4 carriers (44.3% vs. 4.2%) and older participants aged ≥ 60 years (39.8% vs. 40.9%) compared to the low PRS group, which may have further increased observed risk. Other large-scale studies have also demonstrated stratification of dementia risk by PRS. In the Rotterdam study, individuals in the highest quartile of PRS had a 1.85-fold increased risk of dementia compared to the lowest quartile [13]. In Alzheimer’s Disease Neuroimaging Initiative (ADNI), a higher PRS was associated with increased amyloid burden and more rapid memory decline in cognitively normal individuals [15]. Analyses from the UK Biobank excluding APOE from PRS reported hazard ratios between 1.2 and 1.4, highlighting the importance of including APOE to enhance predictive power [16].

We did not find a statistically significant interaction between genetic risk and PA in influencing AD incidence. In fully adjusted models, HRs for high vs. low PA were comparable across genetic risk strata, and interaction terms were non-significant on both multiplicative and additive scales. This finding is consistent with prior large studies using questionnaire-based PA, which also reported independent effects of genetics and lifestyle [11]. Our results further support the observation that PA is beneficial regardless of genetic risk status. In contrast, some smaller studies suggested a stronger benefit of PA among APOE ε4 carriers [17]. These findings suggested that APOE ε4 carriers might derive greater benefit from exercise. However, other studies failed to replicate a significant modification effect or even suggested the opposite pattern [15, 17]. Differences in PA assessment methods and study design may account for the observed inconsistencies. The results of the sensitivity analysis, which employed the IPAQ questionnaire and are presented in the Supplementary Table 1, lend support to our hypothesis. In this study, the use of accelerometry allowed more accurate and objective exposure classification.

PA was protective across all PRS quintiles and APOE genotypes, though the magnitude of effect appeared attenuated at higher genetic risk. The most pronounced risk reductions were observed in individuals within the lowest-to-moderate genetic risk range, while those with APOE ε4 homozygosity or in the top PRS quintile still benefited from high PA, but without fully offsetting their elevated absolute risk. Importantly, no subgroup showed a preference for PA. Previous large-scale studies have similarly reported that healthy lifestyle factors, including regular PA, are associated with reduced dementia risk even among individuals with high genetic risk. For example, one UK Biobank analysis found that high-risk individuals adhering to a healthy lifestyle had approximately 32% lower risk of dementia compared to those with unfavorable lifestyles [11]. Other analyses of UK Biobank participants found similar attenuation of benefit in APOE ε4 carriers [25].

We observed a steep decline in AD risk with increasing PA up to approximately 40 mg units, beyond which the effect plateaued. A data-driven cutoff of 21.7 mg was identified as the optimal threshold for reduced risk, which is below the median value of our sample. This threshold corresponds to relatively low-intensity daily activity. Risk reduction was observed even at levels below the World Health Organization recommendations of at least 150 min of moderate-intensity aerobic activity per week, which is roughly equivalent to achieving an accelerometer count above 30 mg on a daily average basis for older adults [18]. These findings are consistent with research showing that light-intensity physical activity is linked to higher brain volumes and slower brain aging, even in individuals not meeting formal activity guidelines. Meta-analyses have similarly shown increased dementia risk with sedentary behavior and benefits from even modest activity [22]. While higher-intensity exercise may yield added metabolic benefits, our results support promoting accessible activities such as walking or household chores to reduce dementia risk. Multi-domain trials have demonstrated that even moderate lifestyle changes improve cognition in at-risk older adults [10]. On the other hand, the results were less clear when evaluated by questionnaire (Table 5). A previous study evaluating telomere length also demonstrated that measurement by accelerometer yielded more accurate results [20]. Consistent with the findings of the preceding study, the physical activity levels measured by accelerometer in the present study more precisely reflected risk reduction.

PRS is a combined score of multiple common genetic variants that raise the risk of AD. Many of these genes are involved in inflammation, fat processing, and cellular uptake [1]. High PRS has been associated with faster hippocampal atrophy, greater amyloid accumulation, and more rapid cognitive decline, even in preclinical stages [13]. In contrast, physical activity promotes neuroplasticity, elevates brain-derived neurotrophic factor (BDNF), improves cerebrovascular health, and lowers inflammation and oxidative stress [8]. Imaging studies show that physically active individuals tend to have larger hippocampal volumes and slower structural brain decline, including among those at elevated genetic risk [13, 25]. Exercise has also been linked to preserved memory over time in APOE ε4 carriers [17]. These findings suggest that genetic and lifestyle factors influence AD risk through complementary mechanisms, and that fitness may help build cognitive reserve, supporting brain function and delaying clinical symptoms even in the presence of underlying pathology.

Key strengths of this study include the large sample size of over 93,000 participants providing excellent statistical power and objective measurement of physical activity using wrist-worn accelerometers, overcoming limitations of self-reported data. This is among the first studies to examine dementia risk with device-measured activity in such a large cohort. We utilized comprehensive genomic data with a state-of-the-art polygenic risk score for AD [4], making this among the largest studies to jointly analyze accelerometer-measured physical activity and polygenic risk for dementia. The prospective design with median 15.5-year follow-up minimizes reverse causation concerns.

Several limitations should be acknowledged. UK Biobank participants tend to be healthier, more educated, and predominantly White European, limiting generalizability and resulting in low dementia incidence of 0.4%. AD ascertainment relied on electronic health records, potentially missing milder cases and allowing misclassification even though we used ADO classification [26]. Accelerometry data were collected only at baseline for 7 days, and changes during follow-up could not be captured, potentially attenuating associations. Residual confounding by unmeasured factors remains possible. Despite these limitations, our study provides robust observational evidence leveraging objective measurements and genetic stratification. It is known that factors, such as sleep and nutrition, strongly influence AD [19, 21]. In this study, sleep duration was controlled as a binary variable, but nutrition was not. Further studies are needed to examine these variables in more detail alongside PA. Our polygenic risk score included APOE variants, which are the strongest known genetic determinants of Alzheimer’s disease risk. While we adjusted for APOE ε4 carrier status as an independent covariate in all models, the PRS effect estimates may still be substantially influenced by APOE. Future studies utilizing APOE-excluded polygenic risk scores would help to better disentangle the independent contributions of APOE versus the broader polygenic background, and to determine whether gene-environment interactions differ between these genetic components. Nonetheless, our finding that physical activity confers protection across both APOE ε4 carrier status and PRS levels suggests broad applicability of this lifestyle intervention regardless of specific genetic architecture.

In conclusion, higher PA is associated with lower Alzheimer’s disease risk, independent of polygenic and APOE-derived genetic risk. Genetic risk and lifestyle factors contribute independently to AD risk, meaning that physical activity confers protective benefits at every level of inherited risk. While the protective effect may be attenuated in individuals with high genetic risk, it remains significant. Our analysis indicates that meaningful benefits are achievable with relatively low-intensity activity, supporting physical activity as a broadly applicable preventive strategy for AD. Public health recommendations encouraging even light-intensity movement may be particularly important considering the lack of widely accessible disease-modifying treatments and the increasing burden of AD.

## Electronic supplementary material

Below is the link to the electronic supplementary material.

Supplementary material 1 (DOCX 19 kb)

## Data Availability

The data used in this study were obtained from the UK Biobank under application number 435869. Access to the data is available to researchers upon approved application at https://www.ukbiobank.ac.uk.
