# Supplementary material for: Physical activity and Alzheimer’s disease risk across genetic susceptibility: a prospective UK Biobank study using accelerometer data
Source: J Neurol. 2025 Dec 2;273(1):1. doi: 10.1007/s00415-025-13524-z (PMC12672744; doi:10.1007/s00415-025-13524-z)
Supplement: Supplementary file 1 — Supplementary material 1 (DOCX 19 kb) [file 415_2025_13524_MOESM1_ESM.docx]

Supplementary Table 1. Risk of Alzheimer's disease according to physical activity assessed using IPAQ questionnaires based on PRS quartiles and APOE genotype

| (a) Adjusted* Hazard Ratios (HRs) and 95% Confidence Intervals by Combined Exposure Groups | | | |
| --- | --- | --- | --- |
| Group in APOE ε4 non-carrier | HR [95% CI] | Group in APOE ε4 carrier | HR [95% CI] |
| PRS High +High PA (ref) | 1.000 (ref) | PRS Low + PA High (ref) | 1.000 (ref) |
| PRS High +Low PA | 0.722 [0.375–1.393] | PRS Low + PA Low | 0.635 [0.123–3.277] |
| PRS High +High PA | 3.252 [1.998–5.293] | PRS High + PA High | 1.479 [0.596–3.670] |
| PRS High +Low PA | 2.777 [1.622–4.756] | PRS High + PA Low | 1.532 [0.610–3.845] |
|  |  |  |  |
| (b) Additive Interaction Indices |  |  |  |
| Index | Estimate [95% CI] | Index | Estimate [95% CI] |
| RERI | -0.197 [-2.426, 2.032] | RERI | 0.418 [-1.791, 2.627] |
| Attributable Proportion (AP) | -0.071 [-0.874, 0.732] | Attributable Proportion (AP) | 0.273 [-1.179, 1.725] |
| Synergy Index (SI) | 0.900 [0.082, 9.882] | Synergy Index (SI) | 4.683 [0.026, 828.998] |
| *Adjusted for age, sex, Townsend depriviation index, ethnicity, education, smoking status, alcohol status, sleeping time per day, body mass index, and prevalence of hypertension/diabetes | | | |
